# How Can We Make Active Learning Work in K–12 Education? Considering Prerequisites for a Successful Construction of Understanding

**DOI:** 10.1177/1529100621997376

**Published:** 2021-04-19

**Authors:** Garvin Brod

**Affiliations:** 1 Leibniz Institute for Research and Information in Education (DIPF), Frankfurt am Main, Germany; 2 Department of Psychology, Goethe University, Frankfurt am Main, Germany

Active learning holds great promise for improving education, particularly in science, technology, engineering, and mathematics (STEM). Instead of receiving information passively, students take agency and actively construct their own understanding. A large meta-analysis has suggested that these features improve student performance in STEM (Freeman et al., 2014). Many instructional practices that promote active learning have the added benefit of making students familiar with the scientific process of testing theories via predictions and observations. Active learning could also contribute to reducing achievement gaps and empowering students from underrepresented groups to consider careers in science. It therefore seems paramount to synthesize a framework of active learning that guides research and practice in this field, and I applaud Lombardi and colleagues (this issue) for their interdisciplinary efforts to do so.

Although the promises of active learning are wide-ranging, research on its merits has predominantly focused on undergraduate instruction. The meta-analysis by Freeman and colleagues (2014) focused exclusively on undergraduates, and so does the synthesis by Lombardi and colleagues. Does active learning work equally well for younger students, from kindergarten to 12th grade (K–12)? Or are there prerequisites for benefiting from active learning that younger students do not yet meet? And can the construction-of-understanding ecosystem proposed by Lombardi and colleagues inform research and practice in K–12 education as well?

Answers to these questions are important for improving scientific literacy in society at large. Attempting to close achievement gaps at earlier ages is more effective and has higher returns than doing so later (Heckman, 2006). Furthermore, bringing active-learning practices into K–12 education could facilitate the transition to such practices at the undergraduate level. Currently, active-learning methods are often less popular among first-year undergraduate students than among more advanced undergraduates who have more experience with these methods (Zinski et al., 2017). Therefore, in the following, I attempt to provide some answers, acknowledging that these are preliminary and subject to future research that will hopefully be sparked by the construction-of-understanding ecosystem framework. Leaning on the synthetic definition offered by Lombardi and colleagues, I use *active-learning practices* as an umbrella term for instructional activities that are intended to afford students agency over their learning and that foster active construction of understanding.

Do active-learning practices work as well in K–12 education as in undergraduate instruction? This question turns out to be surprisingly difficult to answer. A first difficulty is terminology. Most research that has dealt with active-learning practices in K–12 education has placed them under the umbrella term *inquiry-based teaching/learning*, which spans even wider than active learning. Putting terminology issues aside for a moment, several fairly up-to-date meta-analyses have contrasted some forms of active-learning practices as realized under inquiry-based curricula with more directive instructional practices (Alfieri et al., 2011; D’Angelo et al., 2014; Furtak et al., 2012; Lazonder & Harmsen, 2016; Rönnebeck et al., 2016). Findings regarding effectiveness of these curricula in improving student performance have been mixed and underwhelming. A common thread across these meta-analyses is that effectiveness is higher (a) when learners receive more guidance and (b) when learners are older (i.e., in higher grades). The amount of guidance provided and students’ age are inversely correlated, however, which means that the number of studies in which younger students have received low guidance is small. Furthermore, researchers and practitioners can be expected to tailor task demands to students’ age (Lazonder & Harmsen, 2016). Taken together, these factors render it difficult to interpret age-related differences in the effectiveness of active-learning practices as revealed by meta-analyses.

If not with meta-analyses, how can we move forward in understanding whether, how, and for whom active-learning practices work? I think that the construction-of-understanding ecosystem can be helpful here because it facilitates the identification of common prerequisites for benefiting from active-learning practices. Concisely, as illustrated in Figure 1a, the framework assumes that active-learning practices afford students agency over their learning, which involves their having to make sense of data and observations themselves. Sense making, as reflected by the four upper boxes, involves integrating data and observations with prior knowledge and scientific models. From a psychological perspective, we are talking about the effects of (a) agency beliefs, (b) prior-knowledge activation, (c) cognitive capacities for sense making, and (d) metacognitive capacities for self-reflection. As briefly summarized in the next section, all of these psychological constructs have been intensively researched, and their developmental trajectory is well known (for a more detailed account, see Brod, 2020). This summary provides the basis for the quintessence of this commentary, which is that knowledge of developmental trajectories and the effects they have on learning can guide our understanding of how to make active-learning practices work in K–12 education and beyond.

**Fig. 1.**
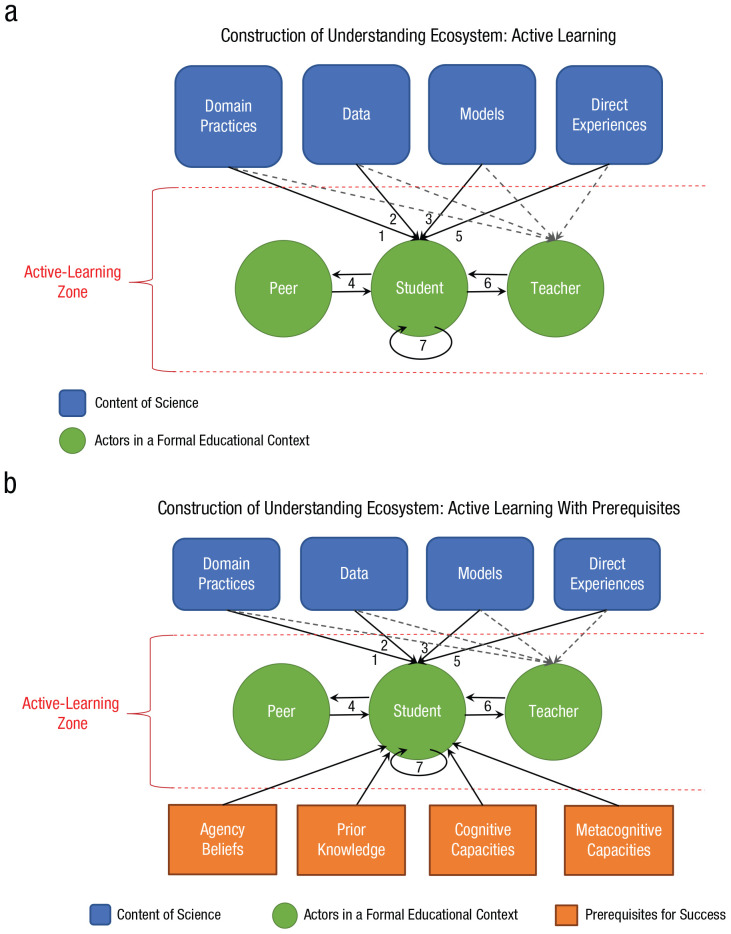
A modification of Lombardi and colleagues’ construction-of-understanding ecosystem. The original framework is shown in panel (a); the modified version, shown in panel (b), includes prerequisites for benefiting from active-learning practices. These four psychological constructs have been shown to determine how strongly students can benefit from active learning and are thus targets for providing support to students.

## Prerequisites for Active Learning Develop Across Childhood

Agency beliefs in the context of learning refer to the perceived capacity to exercise control over one’s learning, and they are positively related to students’ motivation and learning outcomes (Bandura, 2006; Cordova & Lepper, 1996). The relation between students’ agency beliefs and their performance in cognitively challenging tasks has been shown to increase across elementary and early secondary school (Chapman et al., 1990). A recent experimental study investigated how the ability to actively control study affected later memory performance among kindergarten and elementary-school children (Ruggeri et al., 2019). It found that the benefit of giving learners control over their studying emerged around the age of 6 and continued to increase across the elementary-school years. In sum, developmental psychological research suggests that the benefits of agency increase at least until the early secondary-school years.

The amount of relevant knowledge that students already have in a particular domain (i.e., their prior knowledge) is a strong predictor of learning performance and later memory (Schneider, 1993; Shing & Brod, 2016). High levels of prior knowledge facilitate elaboration and organization of to-be-learned material (Schneider, 1993). Students’ general knowledge and their age are closely correlated (Li et al., 2004). Levels of prior knowledge are a strong predictor of how much students will benefit from learning activities in which they have to construct understanding themselves, as is the case in many active-learning practices; whereas students with high levels of prior knowledge can benefit, students with little prior knowledge often struggle (Kirschner et al., 2006). The available evidence thus suggests that students with lower levels of prior knowledge will benefit less from active-learning practices, which often (though not always; see Chi, 1978) puts younger children at a disadvantage.

As detailed by Lombardi and colleagues, understanding whether data match a scientific model requires cognitive capacities, particularly analogical reasoning skills. Analogical reasoning, in turn, relies heavily on working memory capacity (Simms et al., 2018). Both analogical reasoning and working memory capacity exhibit strong and largely parallel age-related improvements that continue across the secondary-school years (Luna et al., 2004; Richland et al., 2006). A recent study demonstrated that children’s analogical reasoning capacities are a strong predictor of how much they will profit from actively generating examples of abstract concepts (Breitwieser & Brod, 2021). Coming up with a good example of an abstract scientific concept requires analogical reasoning because students have to compare potential examples to find the best match to the scientific model (Duit, 1991). Evidence thus suggests that students with higher cognitive capacities will benefit more from active-learning practices than students with lower cognitive capacities. This pattern again speaks for an age-related increase in the extent to which students benefit from many active-learning practices.

Self-reflection refers to the evaluation of one’s current internal status and allows students to initiate corrective adjustments during learning. It is thus a metacognitive process (Lyons & Zelazo, 2011). The accuracy of metacognitive judgments improves substantially across the elementary- and secondary-school years (Roebers, 2017; Schneider, 2008). Children’s growing ability to accurately monitor their current learning activities is coupled with improvements in strategic learning behavior and performance (for a review, see Schneider, 2010). Sufficiently developed metacognitive capacities are necessary for various aspects of strategic learning behavior required for many active-learning practices, including allocating study time on the basis of task difficulty (Lockl & Schneider, 2003; Metcalfe & Finn, 2013) or using organizational learning strategies (Grammer et al., 2011). In sum, evidence suggests that students with higher metacognitive capacities will benefit more from active-learning practices; young children’s immature metacognitive capacities suggest that they will profit less from them.

Taken together, research in developmental psychology suggests that there are prerequisites for benefiting from active-learning practices. Agency beliefs, prior knowledge, and cognitive and metacognitive capacities all determine the benefits of active-learning practices. All of these prerequisites exhibit an increase in functioning across childhood, albeit not all at the same pace, which has an effect on how much children of a specific age can profit from various active-learning practices.

## How Considering Prerequisites for Active Learning Could Guide Its Use in K–12 Education

What does the evidence on prerequisites for benefiting from active-learning practices mean for the question of whether they can work in K–12 science education? At first glance, the evidence looks bleak. The strong age-related increases in effectiveness observed for all prerequisites suggest that the effectiveness of active-learning practices increases across the K–12 years and is overall lower than for undergraduates. This suggestion is in line with the underwhelming findings from meta-analyses on the effectiveness of inquiry-based methods in K–12 education discussed above. In the following passages, I argue that there is nevertheless reason for optimism, chiefly because knowing about the prerequisites of active-learning practices and their development can lead to using these practices wisely.

First, although it is true that children’s knowledge and abilities continue to grow substantially across K–12, basic forms of all of these prerequisites are in place by the beginning of elementary school. This means that most K–12 students are, in principle, able to benefit from active-learning practices. This point is illustrated by a number of experimental studies that demonstrated enhanced learning among children who exercised some control over their learning (Partridge et al., 2015; Ruggeri et al., 2019), had to come up with solutions themselves instead of being provided with them immediately (Brod et al., 2020; Daubert et al., 2020), or engaged in guided exploration (see Weisberg et al., 2016; Yu et al., 2018). Taken together, these findings suggest that active-learning practices can be used to improve learning outcomes among K–12 students.

Second, K–12 students’ ability to benefit from active-learning practices increases when they are adequately supported. Providing additional guidance, particularly for younger students, can avoid the pitfalls of pure inquiry-based learning approaches, which often work only for students with high prior knowledge (Kirschner et al., 2006). As found in the meta-analysis by Lazonder and Harmsen (2016), providing guidance improves K–12 students’ learning outcomes considerably across various practices and STEM domains. Effective ways to do so include prompting students to perform a specific action, providing them with some parts of the required action or solution (i.e., scaffolding), or teaching them about useful strategies (e.g., Kuhn et al., 2000). Why not provide guidance for all students all the time, then? Studies on guidance during learning suggest an inverted-U-shaped relation between task difficulty and learning success, which indicates that high levels of guidance can harm students with high knowledge or abilities (Koedinger et al., 2008). Active learning with little guidance can thus be thought to introduce a desirable difficulty for students (Kachergis et al., 2017). The key challenge for teachers in K–12 education is therefore to determine the right level of guidance for students engaged in active-learning practices. If guidance is provided at an appropriate level, active learning can prove effective throughout K–12.

Third, knowledge of prerequisites for active learning can help instructors choose those active-learning practices that are likely to work best for a particular group of students and to provide the right level of guidance. In a recent study that illustrates this approach (Breitwieser & Brod, 2021), late-elementary-school children and undergraduates studied science facts using either of two active-learning practices: They had to either generate a prediction or come up with an example before seeing the correct information (a numerical fact). These two active-learning practices were contrasted because their cognitive prerequisites are known to differ. In particular, generating a good example for a scientific concept requires well-developed analogical reasoning skills (Duit, 1991), which are far from mature in late-elementary-school children (Richland et al., 2006). Knowledge of prerequisites thus suggests that although generating examples can work well for undergraduates, it should be rather ineffective for elementary-school children. In keeping with this hypothesis, results showed that elementary-school children learned less when they generated examples than when they generated predictions, whereas undergraduates learned comparably well in both conditions (Breitwieser & Brod, 2021). The difference in effectiveness between generating predictions and examples was related to children’s reasoning abilities, in line with its assumed role as a prerequisite. In sum, the findings of this study show that although both generating predictions and generating examples are effective in undergraduates, only generating predictions is effective in elementary-school children because most of them struggle with generating good examples.

Knowledge of prerequisites and their development can further explain successes and failures of various active-learning practices in the classroom. On the one hand, the findings of Breitwieser and Brod (2021) suggest that engaging students in prediction-observation cycles is an active-learning practice that is likely to work already in elementary school. In line with this claim, prediction-observation cycles form part of many successful STEM curricula that have proved effective for elementary- and secondary-school students (e.g., Hardy et al., 2006; Liew & Treagust, 1995; Monaghan & Clement, 2000). On the other hand, the findings suggest that engaging students in active-learning practices that require advanced analogical reasoning abilities, such as coming up with examples or constructing drawings for an abstract concept, are unlikely to work until late secondary school. In line with this claim, a study in which fourth- and sixth-graders had to construct drawings corresponding to a biology text found no benefit of actively drawing relative to inspecting provided drawings in a later problem-solving test (Van Meter et al., 2006). Whereas additional guidance boosted problem-solving performance among the sixth-graders, this was not the case for the fourth-graders (for similar findings, see Van Essen & Hamaker, 1990). These findings suggest that although older students can benefit from constructing drawings if guidance is high, elementary-school students struggle to benefit from doing so at all, likely because of excessive demands on analogical reasoning.

In sum, knowledge of prerequisites for particular active-learning practices and their developmental trajectory can guide researchers and educators in choosing those practices that are likely to work best for a particular group of students. Exemplary studies detailed above have suggested that some, but not all, active-learning practices work well for K–12 students. This is true particularly for students in lower grade levels, whose cognitive and metacognitive abilities are still undergoing rapid developmental changes. In the final section, I connect this line of research with the construction-of-understanding ecosystem and try to illustrate how together, they can guide the implementation of active-learning practices in K–12 education.

## Conclusions

The construction-of-understanding ecosystem proves particularly useful because it provides an actionable framework of active learning that can be applied across disciplines. An assumption inherent in the framework is that active-learning practices are beneficial for student achievement in STEM. In this commentary, I have argued that there are prerequisites for this benefit to occur. Active-learning practices are demanding in that students need advanced cognitive and metacognitive capacities to be able to profit from them without being closely guided. The effects of insufficient cognitive and metacognitive capacities become most visible in younger students for whom these capacities are still developing. For example, elementary-school students struggle with constructing examples or drawings of abstract concepts as a result of their immature analogical reasoning skills (Breitwieser & Brod, 2021; Van Meter et al., 2006). This should not be taken to mean that active-learning practices cannot be beneficial for K–12 students, however. Prerequisites differ between practices, and a lack of them can often be counteracted with additional guidance. Instead, this commentary is intended as a pledge to consider prerequisites in order to choose those active-learning practices that are likely to work best for a particular group of students.

In line with this pledge, I took the liberty of slightly modifying the construction-of-understanding ecosystem to illustrate how prerequisites for a successful construction of understanding could be included. As illustrated in Figure 1b, students’ agency beliefs, prior knowledge, cognitive capacities, and metacognitive capacities are new nodes that reflect prerequisites for the success of active-learning practices. As detailed above, these four psychological constructs have been shown to determine how strongly students can benefit from active learning and are thus prime targets for providing support to students. The arrows to “Student” reflect this influence. I think that including prerequisites for success serves the purpose of the construction-of-understanding ecosystem, which is to provide an actionable framework of active learning that guides researchers and practitioners. In this spirit, knowledge about prerequisites for success can guide both researchers and practitioners in tailoring active-learning practices to the needs of their students.

As a final remark, it is important to note that considering the typical developmental trajectory of a particular prerequisite can provide only a rough heuristic for deciding which active-learning practice to use and how much guidance to provide. Age is only a crude proxy for the status of a student’s cognitive and metacognitive capacities, and there are substantial differences in these capacities among children of the same age. Seen from a different angle, however, this means that considering prerequisites for benefiting from active learning can also improve its success for undergraduates, who differ substantially in these prerequisites as well. Providing additional guidance or withholding it on the basis of students’ prerequisites can improve the effectiveness of active learning for all students. A distant goal of research on active-learning practices should thus be to tailor their application to each individual learner by taking into account relevant learner characteristics. To achieve this goal, however, much more work is needed on the interplay between active-learning practices, their prerequisites, and the amount of guidance provided. I hope that the construction-of-understanding ecosystem will contribute to sparking this research.

## Transparency

*Editor:* Nora S. Newcombe
